# Education

**Published:** 1881-05

**Authors:** 


### EDITORIAL.

### EDUCATION.

The thorough and systematic training of nurses for the sick is a subject receiving much attention from the medical profession, and of others as well, when they become intelligent upon the subject. So important is this matter, that training schools or colleges have been established, both in this country and Europe, for the proper education of those who take care of the sick.

This is a subject of but little, if any less importance than the training of the physician. In a large per cent. of the cases of sickness, the final result depends quite as much upon the nurse as upon the physician.

In the institutions already established for such education and training there are some noteworthy points; prominent among which are the acquirements for entering, and the natural adaptability for the work. Referring to this matter, Dr. W. G. Wylie, of New York, in his report as one of the Bellevue Hospital Committee on this subject, says, speaking of those about to enter for the course: "First, having made application by answering a form with which she is furnished, she presents credentials, consisting of a note as to *moral character* from a clergyman or other reliable person, and one concerning good health from a physician. The Superintendent examines her in reading, penmanship, arithmetic and English dictation. She is now ready to enter the school for a month on probation. At the end of the month she is allowed to withdraw her application if she so desires, or she may be requested to do so by the Superintendent, should she show mental or physical unfitness for the work. Having given satisfaction, however, during this time, she is now ready for actual work." * * "She is required to study anatomy, physiology and hygiene, to attend lectures on various subjects given by physicians to the class, to be prepared to answer questions on such lectures, and to be thoroughly instructed in the course of training." Following this is given the curriculum, embracing a list of subjects or particulars, twenty-one in number, every one of which is important, and must be thoroughly mastered.

Now this is all well, right and necessary. But then why is it that some such requirements for entrance and continuance in the medical and dental colleges are not made. Why is it that those seeking to enter these training schools, are required to present evidence of moral character, good health and fair education, while those, especially those of the opposite sex, notoriously immoral, profane, vulgar and ignorant, and in some instances standing upon the verge of the grave by their beastiality, are admitted, without let or hindrance, into almost every medical or dental college in the country?

Is it because the former are women? Perhaps not, but rather because of the manifest importance and desirability of having proper qualifications in those who are to nurse and constantly care for the sick. But if this is necessary for those who execute and carry out the orders and directions of the physician, what sort of person ought he to be? If these qualifications and endowments are necessary for those who are to be the nurses for the sick, what should be the qualifications and endowments of those who are to give the orders and directions, which the nurse is to execute, and upon which hang the issues of life and death.

Why do not all medical and dental colleges establish at least as high a standard for those seeking to enter these departments, as are required for those preparing to become nurses.

From the fact that generally *no requirements* are made, except to pay the fees, large numbers, who are totally without adaptation, or interest in the work they undertake, press into these institutions —and large numbers go forth every year, bearing with them the authority of a diploma to practice in some branch of the healing art, whose course will be as certainly that of the empyric, as though they had received their professional education from a "*natural bone setter*," or from the veritable *old Dr. Jacob Townsend.*

Now will not our medical and dental colleges discriminate at least in favor of decency, and a respectable degree of natural and acquired fitness for entering upon a course of professional study.

